# Maternal weight status and responsiveness to preterm infant behavioral cues during feeding

**DOI:** 10.1186/s12884-017-1298-4

**Published:** 2017-04-11

**Authors:** Evanthia A. Arianas, Kristin M. Rankin, Kathleen F. Norr, Rosemary C. White-Traut

**Affiliations:** 1 Department of Women, Children and Family Health Science, University of Illinois at Chicago, College of Nursing, (MC 802) 845 South Damen Avenue, Chicago, IL 60612-7350 USA; 2 Division of Epidemiology and Biostatistics, University of Illinois at Chicago, School of Public Health, Chicago, IL USA; 3 Department of Nursing Research, Children’s Hospital of Wisconsin, Milwaukee, WI USA

**Keywords:** Preterm infants, Maternal weight status, Maternal responsiveness to preterm infant feeding

## Abstract

**Background:**

Parental obesity is highly predictive of child obesity, and preterm infants are at greater risk of obesity, but little is known about obese and non-obese mothers’ responsiveness to preterm infant cues during feeding. The relationship between maternal weight status and response to preterm infant behavioral cues during feeding at 6-weeks corrected age was examined.

**Methods:**

This secondary analysis used data from a randomized clinical trial. Maternal weight was coded during a play session. Mother-infant interaction during feeding was coded using the Nursing Child Assessment Satellite Training Feeding Scale (NCAST). We used multivariate linear regressions to examine NCAST scores and multivariate logistic regressions for the two individual items, satiation cues and termination of feeding.

**Results:**

Of the 139 mothers, 56 (40.3%) were obese, two underweight women were excluded. Obese mothers did not differ from overweight/normal weight mothers for overall NCAST scores, but they had higher scores on response to infant’s distress subscale (mean = 10.2 vs. 9.6, *p* = 0.01). The proportion of infants who exhibited satiation cues did not differ by maternal weight. Obese mothers were more likely than overweight/normal weight mothers to terminate the feeding when the infant showed satiation cues (82.1% vs. 66.3%, *p* = 0.04, adjusted OR = 2.31, 95% CI = 0.97, 5.48).

**Conclusions:**

Limitations include lack of BMI measures and small sample size. Additional research is needed about maternal weight status and whether it influences responsiveness to preterm infant satiation cues. Results highlight the need for educating all mothers of preterm infants regarding preterm infant cues.

**Trial registration:**

NCT02041923. Feeding and Transition to Home for Preterms at Social Risk (H-HOPE). Registered 15 January 2014.

## Background

Obesity is the most common health problem facing both adults and children in the U.S.A today and rates continue to rise [1, 2]. The most recent data from the National Health and Nutrition Examination Survey III suggest that 11% of children and adolescents are obese and an additional 22% are overweight [1]. Parental obesity is highly predictive of later child obesity but the etiology is not clear; may be partly genetic and epigenetic, may relate to role-modeling of eating and exercise [3, 4]. Preterm infants have a higher risk of childhood obesity than infants born at full term, which may be partly related to mothers’ continued perception of frailty and underweight [3, 5, 6]. There is some evidence that obese mothers have feeding practices in early childhood that may contribute to later child obesity [4, 7]. However, almost nothing is known about whether there is a relationship between obesity and feeding practices in early infancy. This secondary analysis takes advantage of a unique opportunity to examine whether obese mothers of preterm infants are more likely to not recognize satiation cues and to continue feeding in early infancy (6 weeks Corrected Age, CA). In this paper we explore this possible link between maternal obesity and inappropriate feeding of their preterm infants.

### Preterm Infants are at higher risk for obesity

Preterm infants are at higher risk for developing obesity than infants born at term [3, 5, 6]. Specifically, they have a larger childhood and adult body size and body mass index (BMI) [8]. One reason preterm infants may be at a higher risk of obesity in later life may be their early feeding difficulties and the parents’ response to these [9]. Preterm infants born before 32 weeks gestation do not yet have the capacity to coordinate sucking, swallowing, and breathing, so their initial intake is through gavage feedings [10–12]. They have unclear behavioral states and cues and are often in a drowsy state where they are likely to not be awake for feedings [10, 11, 13]. Neonatal medical conditions may present additional difficulties in acquiring feeding skills during the first four months. For some infants, their medical conditions delay their opportunities to learn the skills associated with new feeding modalities [2, 14].

The parents’ perception of their preterm infant as medically vulnerable may also contribute to child obesity [7, 9]. Higher parental perception of child vulnerability is associated with poorer developmental outcomes for preterm infants [9, 13, 15–17]. Related to this perceived infant vulnerability, mothers of preterm infants may try to help the infant gain weight by over-feeding the infant [4, 7]. This perception may contribute to mothers overfeeding and not responding appropriately to satiation cues. Mothers often feel that by feeding their child more, the infant will grow at a faster rate [4].

### The importance of infant behavioral cues and maternal responses during feeding

Infant feeding and behavioral cues during feeding are critical in the development of the early mother-infant relationship and later social, language, and cognitive development [13, 18]. This is especially important for preterm infants, who are at higher risk of developmental delays [18]. Building positive patterns of mother-infant interaction requires sensitive maternal responses to infant cues, including reading and responding to distress and specific hunger and satiation cues. Many factors influence the development of the mother’s responsiveness to her infant’s behavioral cues during feeding and the mother-infant relationship. These include maternal age, parity, education, and social support [18]. However, little is known regarding whether maternal sensitive responsiveness during feeding for preterm infants is related to maternal characteristics such as maternal weight status.

### Maternal obesity may affect responses to infant behavioral cues during feeding

Maternal obesity has increased rapidly over the past 20 years and is an important predictor of childhood obesity [19]. Currently more than half of pregnant women are overweight or obese [2, 20]. Women of lower socioeconomic status are more likely to be obese [21, 22].

Children whose parents are obese are four times more likely to be obese themselves than children whose parents are normal weight [23]. The influence of parental obesity on childhood obesity most likely results from a mixture of environmental and genetic influences [23]. Children as young as three years old demonstrate increased preferences for foods high in fat if their parents are obese [23]. Mothers directly influence the child’s eating and exercise habits, and these influences are potentially modifiable. Several studies comparing the infant behavioral cues during feeding of obese and non-obese mothers have found that mothers who were obese were more likely to overfeed their infants [23]. However, there are no studies of responsiveness to early behavioral cues during feeding and maternal weight for mothers of preterm infants, a group that already has an elevated risk of obesity.

The relationship between the mother’s obesity and child obesity may begin developing in early infancy due to poor infant behavioral cues during feeding, such as failing to recognize infant hunger and satiation cues and failure to respond appropriately [3]. One plausible contributing factor is that mothers who are obese, many of whom may have difficulty recognizing and responding appropriately to their own satiation cues, may either fail to recognize their infant’s satiation cues or fail to respond appropriately to those cues by ending the feeding.

Non-obese mothers may be more likely to end the feeding appropriately when the infant appears satiated. However, no prior studies have examined the relationship of maternal obesity with preterm infant behavioral cues during feeding.

To fill this gap, the purpose of this study was to explore the relationship between maternal obesity and preterm infant behavioral cues during feeding when the infant reached 6-weeks corrected age (CA).

## Methods

### Design

This study is a secondary analysis using selected data from a larger randomized controlled trial that tested an integrated intervention for mother-preterm infant dyads at high social-environment risk [24–26]. The intervention, called H-HOPE (Hospital to Home: Optimizing the Preterm’s Environment), included an infant-directed multi-sensory intervention and mother-directed guidance regarding preterm infants, especially behavioral states and cues [24]. Our cross sectional analyses used data from the 6-week CA follow-up visit of the larger study.

### Sample and setting

Mother-infant dyads were recruited from two inner-city community-based hospitals, one with a Level II Neonatal Intensive Care Unit (NICU) that had extended capabilities, and one with a Level III NICU. The selection criteria for the overall trial were: otherwise healthy infants, born at 29–34 weeks gestational age at birth, and whose mothers had at least two of the following social-environmental risk factors: self-identity as Black or Latina, less than high school education, less than 18 years of age, history of or current mental illness, family income less than 150% of the poverty line, more than one child under 24 months, four or more children under age 18 in household, and/or residing in a disadvantaged neighborhood. The study population was not representative of all preterm infants; as mother-infant dyads were recruited at only two hospitals in the inner city of a large Midwestern urban area. In 2015, preterm birth affected 1 out of 10 infants born nationally; non-Hispanic African American women have a higher preterm birth rate of 13%, which non-Hispanic white women have a lower rate of 9% [27]. This sample was likely to have a higher proportion of obese mothers than the general population; as already noted, obesity rates are higher for women from socioeconomically disadvantaged groups [21, 22].

Participants for this sub-study were 139 mothers and infants who completed the 6-week CA follow-up visit and had valid data for the independent and dependent variables of interest. The larger study recruited a total of 230 mother-infant dyads, 32 of which became ineligible after enrollment due to infant health conditions. Of the remaining 198 mother-infant dyads, 149 (75.3%) were retained for the 6-week CA visit, with 142 having valid data for outcome of interest, mother-infant interaction during feeding. Some dyads completing the visit had missing data for this analysis due to an inability to complete the feeding due to infant fatigue, fussiness or lack of readiness to eat or technical malfunction of the audio recording on some videos of mother-infant feedings. One additional dyad was dropped because there was no video-recorded assessment of mother-infant interaction during play, which was the segment of the video used to code maternal weight status. Two more dyads were dropped from analysis because the mother was underweight, and there was not a large enough sample of underweight mothers to examine the association of underweight and infant behavioral cues during feeding. The final analytic sample size was 139.

## Measures

### Maternal weight status

**Fig. 1:**
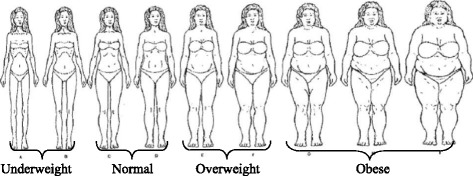
Culturally Relevant Body Image Instrument, Pulvers et. al. [22]: validated instrument

Since this secondary study used data from a larger trial with a different aim, there was no direct measurements or self-reports of the mother’s weight and height at any time from pre-pregnancy through the 6-week CA visit, making it impossible to calculate BMIs for participants. We used video-recorded play sessions with the infant, when mothers were standing, usually in profile, to code mothers’ approximate BMI category. We used a previously developed validated culturally-appropriate pictorial instrument (Fig. 1) [22] designed to be used by an observer. The pictorial instrument was developed using non pregnant African American women. The pictorial representations were validated in the original study by examining correlations between ratings of three different observers and actual BMI determined from measurements of height and weight. The correlations between the ratings and BMI were uniformly high, ranging from 0.82 to 0.93 for the three observers [28]. As shown in Fig. 1, these pictures correspond to the CDC weight categories. A body mass index (BMI) of 18.5 or less is considered underweight, a BMI of 18.5-24.9 is considered normal, a BMI is 25–29.9 is overweight, and a BMI of 30 or over is obese. Women labeled A and B were coded as underweight, the women labeled C and D were coded as normal weight, the women labeled E and F were coded as overweight, and the women labeled G, H, and I were coded as obese. Since only two women were coded as underweight, the underweight women were excluded from the sample for this study. We dichotomized the ratings as non-obese (normal and overweight) or obese. We randomly selected 30% of the cases (*n* = 42 of 139) and these were re-rated for weight status by a second trained observer, with 97.7% agreement (Kappa = 0.95), indicating high agreement [29].

### Mother-infant interaction and infant behavioral cues during feeding

Maternal and infant behaviors during feeding at 6-weeks CA were assessed using the Nursing Child Assessment Satellite Training-Feeding Scale (NCAST-Feeding Scale) [30]. The NCAST-Feeding Scale (76 items, 1 point for each item, range 0–76) consists of six subscales: (a) maternal sensitivity to cues; (b) maternal response to distress; (c) maternal social-emotional growth-fostering; (d) maternal cognitive growth fostering; (e) infant clarity cues; and (f) infant responsiveness to caregiver which are summed to yield a mother scale (a-d), an infant scale (e-f), and a total scale. In the total and sub score, a higher score indicates more positive mother-infant interaction. The NCAST-Feeding Scale has been widely used in parenting research to measure the overall quality of the mother-infant interaction and has well established reliability (α = .86 for the total scale and .83 for the mother score and .73 for the infant score) and predictive validity (significantly correlated with social and cognitive development scores at 24 and 36 months) for term and preterm infants [1, 31]. For this study, we examined the total score and the four mother subscales. To focus specifically on overfeeding, we also examined two individual items: “infant demonstrates satiation cues,” Item 64 from the infant clarity of cues subscale, and “mother terminates the feeding when the child shows satiation cues,” Item 14 from the sensitivity to cues mother subscale. Terminating feeding in response to satiation cues is a positive maternal behavior.

The feeding interactions were video-recoded and later judged by a coder. Two coders were trained to criterion of > 90% agreement. Inter-rater reliability between the primary and a secondary coder remained above 95% agreement. Both coders were blinded to the infant’s group assignment and to the idea for this secondary analysis. While they could see the mother’s weight status in the video, coding was completed prior to the idea of conducting this secondary analysis. Coders had no idea that there might be an analysis looking at maternal weight status.

### Covariates

Maternal baseline characteristics used as covariates included age, race/ethnicity (African-American or Latina), education appropriate for age, parity, annual household income above, and whether in the intervention or control group. Because some participants were adolescents, education was categorized as low for women 20 or older who did not have a high school degree or GED, and for women <20 who did not finish high school or were not currently still in school.

Annual income was dichotomized to < 185% federal poverty level (FPL) or ≥ 185% of the FPL, the cutoff in this state used to determine eligibility for the Women, Infant, and Children (WIC) nutrition program. Women are carefully screened by WIC to determine their income eligibility, so this is an accurate measure of income. In an urban environment, 185% of the FPL is generally regarded as a threshold for income adequacy [32, 33].

Although women in the RCT were randomly assigned to the intervention or an attention control group, the intervention did not relate to the outcomes of interest and was not included as a covariate in these analyses. We conducted regressions with and without the intervention and adding the intervention made no difference in the results. Therefore, we did not include intervention in our final regression models.

## Procedure

Prior to recruitment, this study was approved by the Institutional Review Board for the University of Illinois at Chicago and the two hospital sites. The in-hospital research nurses confirmed infant and maternal eligibility. Mothers and their infants were enrolled by a member of the research team after confirmation of infant’s health, mother’s eligibility, and mother’s willingness to participate. After informed consent was obtained and prior to assignment to the intervention or control group, an in-person baseline interview was conducted with the mother. Interview data, medical record review, and biologic measures were obtained at baseline and during the hospital stay. The NCAST-Feeding was assessed at hospital discharge and at a 6-week CA follow-up visit. Only the latter administration of the NCAST-Feeding was used for this study.

### Statistical analysis

Univariate statistics were used to describe the sample. Using *t*-tests or chi-square tests, we examined the crude relationship between the independent variable and each of the dependent variables, as well as the relationship of each covariate with the independent and dependent variables. As discussed earlier, because the intervention had no relationship with the dependent variable we did not use this as a covariate. Multivariable linear regression models were used to obtain adjusted estimates of the difference in mean NCAST scores for each scale (total scale and four maternal subscales) for obese vs. overweight/normal weight mothers (termed non-obese), after adjusting for all maternal and infant covariates described in measures. Multivariable logistic regression models were run to obtain a crude and adjusted odds ratio (95% confidence interval) for the relationships between maternal obesity and each of the two individual NCAST items: infant displays satiation cues and appropriate termination of feeding by the mother [34]. For all models, manual backward elimination of covariates was employed and those that were not significantly related to the outcome were dropped from the final models presented here.

## Results

One hundred thirty-nine mother-infant dyads had complete data and were included in this analysis. Fifty-six mothers (40.3%) were classified as obese, 54 (38.8%) overweight, and 29 (20.9%) normal weight. Since outcomes were similar for overweight and normal weight mothers, they were combined for analysis into a non-obese group.

Maternal and infant characteristics for this sample are shown in Table 1. The mean maternal age was 26.3 (range = 15–45) and 18.7% of the mothers were under 20 years old. More than half of the participants were multiparous (60.4%). Approximately half of the women identified themselves as African American and half as Latina. The majority of participants (76.3%) were educated appropriately for their age (either completed high school or its equivalent or still in school). Most participants (88.2%) were below 185% of Federal Poverty level. About half of the mothers were in the H-HOPE intervention group.

**Table 1:** Sample Characteristics, by Weight Status and Overall

Value		Obese (n = 56)	Non-obese (n = 83)	Overall (n = 139)
Mother				
Age*	mean (SD)	28.0 (6.4)	24.7 (6.4)	26.0 (6.5)
Race/Ethnicity (%)	African-American	55.4	45.8	49.6
	Latina	44.6	54.2	50.4
Education^a^ (%)	Low education	21.4	24.4	23.2
	Appropriate for age	78.6	75.6	76.8
Parity (%)	Primparous	32.1	44.6	39.6
	Multiparous	67.9	55.4	60.4
Income (%)	<185% FPL	85.7	90.0	88.2
	≥185% FPL	14.3	10.0	11.8
Infant				
Sex	Female	44.6	49.4	47.5
	Male	55.4	50.6	52.5
Gestational age	mean (SD)	32.5 (1.7)	32.4 (1.6)	32.4 (1.6)
Birthweight (g)	mean (SD)	1896 (428)	1802 (396)	1839 (410)
Infant morbidity score^b^	mean (SD)	74.6 (3.2)	69.2 (16.9	71.2 (19.9)
Weight at 6 weeks CA^c^ (g)^*^	mean (SD)	5046 (747)	4955 (702)	4997 (715)
Mother-Infant Dyad:				
Intervention Group	H-HOPE^d^	39.3	50.6	46.0
	Control	60.7	49.4	54.0

*p<0.05

*FPL* Federal poverty level

^a^Defined as low education for women 20 or older without a high school degree or GED or women <20 who did not finish high school and are not currently in school; otherwise defined as appropriate for age

^b^4 missing values

^c^Corrected Age

^d^Hospital to Home: Optimizing the Premature Infant’s Environment - Intervention Group

Regarding infant characteristics, about half of the infants were male, their mean gestational age at birth was 32.1 weeks and mean birthweight was 1839 g. They had an average POPRAS morbidity score of 72, reflective of clinical stability in conjunction with premature birth. At the 6-week CA visit, they had a mean weight of almost 5000 g, considered within normal range for infants at this age [35].

Only one of these maternal and infant covariates was significantly different for obese versus non-obese mothers. Obese mothers were older, with a mean age of 28, compared to the mean age of 24.7 for the non-obese mothers.

Table 2 shows the mean NCAST total score and maternal subscales by weight status. Obese and non-obese mothers had similar means for the total score and three of the four maternal subscales. Only one of the maternal subscales, response to infant’s distress, was significantly different for obese versus non-obese mothers (*p* < 0.05). For this subscale, obese mothers had higher responsivity to infant cues than non-obese mothers.

**Table 2:** Mean NCAST Total Score and Maternal Subscales by Weight Status

NCAST Scale	Obese Mothers (*n* = 56) mean (SD)	Non-obese Mothers (*n* = 84) mean (SD)
Full Scale^a^	63.6 (4.9)	63.2 (7.1)
Maternal Subscales^b^:		
Sensitivity to Cues	14.5 (1.4)	14.1 (1.5)
Response to Child’s Distress*	10.2 (1.1)	9.6 (1.5)
Social-Emotional Growth Fostering	12.1 (1.5)	12.1 (2.0)
Cognitive Growth Fostering	7.8 (1.8)	7.6 (2.0)

*p < 0.05

^a^Maximum possible score = 76

^b^Maximum possible scores = 16, 11, 14, and 9, respectively

We then conducted multivariable regression analyses with all covariates for the maternal response to infant’s distress subscale, removing non-significant covariates to produce a final model (Table 3). Maternal weight status remained a significant predictor of maternal response to infant distress after adjustment for significant covariates, with obese mothers demonstrating significantly higher scores on the maternal response to infant’s distress subscale than non-obese mothers (Table 3). In addition to weight status, the only covariates significantly related to maternal response to infant’s distress were education and the infant morbidity score. Mothers with low education for age had significantly lower scores, and mothers whose infants had a higher morbidity score had higher scores (Table 3). However, only a small proportion of the variation in maternal response to infant distress was explained by these three variables in the regression (R^2^ = 0.13).

**Table 3:** Linear regression for relationship between maternal obesity and NCAST—Maternal. Response to infant’s Distress Subscale (n = 134). R^2^= 0.10

Model Parameter	Beta	Standard Error	*p*
Intercept	8.80	.44	<.01
Obese vs. Non-Obese	.60	.24	.01
Low vs. Appropriate Education level^a^	-.65	.27	.02
Infant Morbidity Score (POPRAS)	.01	.01	.03

^a^Education was considered low for women 20 or older without a high school degree or women

<20 without a high school degree and not still attending school

**Fig. 2:**
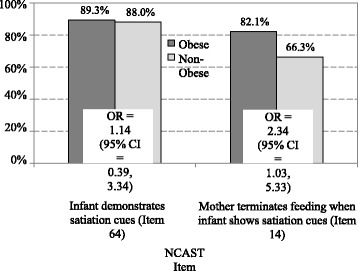
Differences in individual NCAST items about infant satiation cues and maternal responses to those cues for obese and non-obese mothers

We also estimated odds ratios for the relationship between weight status and two specific items observed as part of the NCAST-Feeding: whether the infant demonstrated satiation cues, and whether the mother terminated the feeding in response to infant satiation cues [30]. As Fig. 2 shows, there was no significant difference in the proportion of infants displaying satiation cues for obese compared to non-obese mothers (89.3% vs 88.0%, respectively), but obese mothers were at 2.3 times increased odds (95% CI = 1.03, 5.33) of appropriately terminating the feeding in response to infant satiation cues. We then conducted a multivariable logistic regression with all covariates for terminating the feeding in response to infant satiation cues following the same procedure described above to produce a final model. Table 4 shows the odds ratios for significant predictors in final model. Obese mothers continued to be at significantly increased odds of responding appropriately to infant satiation cues (odds ratio 2.47, CI 1.08 – 5.77). The only other significant predictor was male sex; mothers with male infants were at significantly decreased odds of responding appropriately to infant satiation cues (odds ratio 0.44, CI .0.19 – 0.97).

**Table 4:** Odds Ratios: Logistic regression for relationship between maternal obesity and NCAST Item #14 Maternal Response to Infant’s Satiation Cues (*n* = 139)

Model Parameter	Odds Ratio	95% Wald’s Confidence Limits
Obese vs. Non-Obese	2.49	1.08 – 5.76
Male Infant Sex	0.44	0.19 – 0.97

## Discussion

The purpose of this study was to examine the relationship between maternal obesity and preterm infant behavioral cues during feeding at 6-weeks CA. There were no differences between the obese and non-obese mothers in overall mother-infant interaction scales. However, compared to non-obese mothers, obese mothers were more responsive to cues when their infants were distressed and more often terminated the feeding when their infants showed satiation cues.

Response to infant distress, one of the maternal sub scores of the NCAST, is an important component of building positive mother-infant interactions, and is discussed in measures. Scoring well on responsiveness to distress requires the mother to recognize infant signals of distress and then take actions to alleviate the distress, such as stopping or starting the feeding, changing the baby’s position, and provide soothing verbal or non-verbal efforts. These appropriate maternal responses build the infant’s sense of control and trust that the mother understands and responds to infant needs, an important component in building synchrony and mutual responsiveness.

These findings were surprising because we expected that non-obese mothers would be more likely to respond appropriately to preterm infant distress and satiation cues during feeding than obese mothers. Few studies have examined the relationship between maternal obesity and other aspects of the mother’s feeding behaviors during infancy. One area of previous research has suggested a correlation between maternal high body mass index and early termination of breastfeeding [14, 36–38]. However, the relationship between breastfeeding factors and child obesity is not clear, with contradictory findings across studies [39, 40], so it is not yet clear whether differences in breastfeeding patterns are an important factor in the link between maternal and child weight status. We have not found any prior studies of maternal obesity and responsiveness to infant behavioral cues during feeding, especially for preterm infants.

The prevailing stigmatization and discrimination experienced by persons who are obese in the USA and other European countries is well documented and includes negative attitudes and discrimination in the health sector [41]. Expecting that obese mothers would be less responsive to their preterm infant’s cues during feeding may reflect these stigmatizing attitudes, and at least in this small study the results challenge the idea that maternal obesity is related to non-responsiveness to satiation cues and thus overfeeding in infancy.

In this report, mothers whose infants who had higher illness scores as measured by the POPRAS were more likely to respond to their infant’s distress. This finding could likely be related to mothers compensating for illness severity with an increased response to their infant’s distress. Maternal education also contributed to lower scores on response to infant distress. This finding is similar to previous reports [42]. For the study’s total sample, female infants weighed less at birth yet had a higher mean weight gain from birth through hospital discharge than the male infants [25]. Perhaps the lower weight gain during hospitalization of the male infants influenced mothers to continue feeding their male infants even after they exhibited satiation cues.

In interpreting these study’s results, two major limitations of this study need to be considered. This study lacked a direct measure of maternal BMI, the sample size was relatively small. Therefore, we should be cautious about generalizing beyond this study.

A major strength of this study was the use of a well-established measure of mother-infant interaction during feeding. Videotaping of the feeding allowed repeated viewing as necessary to accurately record mother and infant behaviors and double-coding established high reliability.

Our findings also identified the need to provide more pre-discharge education regarding preterm infants’ behavioral cues during feeding to all mothers of preterm infants, as many non-obese mothers did not recognize and appropriately respond to infant distress or satiation cues. In our overall sample, 27% of mothers did not terminate the feeding appropriately in response to their preterm infants’ satiation cues. Mothers of preterm infants who have low income, lower education, and/or minority status, such as those in our sample, are at a greater risk than their higher income counterparts for poor mother-infant interaction and infant outcomes [24].

## Conclusions

This is the first report of maternal weight status and its potential influence on preterm infant behavioral cues during feeding. In this small study with only an indirect measure of obesity, our findings suggest that obese mothers are equally or more likely to end a feeding when they observe infant satiation cues than normal/overweight mothers. A larger study with direct measures of height and weight should be conducted to provide further evidence regarding the relationship between maternal obesity and responsiveness to preterm infant feeding cues.

Since preterm infants are at higher risk for developing obesity compared with infants born at term, it is important to identify whether the mother’s weight status relates to early overfeeding. These unexpected initial findings suggest that later obesity among preterm infants is not related to early inappropriate responses to infant cues or overfeeding by obese mothers. Future studies are needed examining maternal weight status and behavioral cues during feeding throughout infancy for both premature and full term infants, and how these behavioral cues during feeding relate to childhood obesity. Our findings raise questions about the usual assumption that inappropriate early parental feeding is a major factor explaining the strong correlation between having an obese parent and childhood and adult obesity. It is possible that obese parents’ overfeeding and encouragement of overeating do not begin until the child is older [4, 7], or that parental feeding patterns are less important than other factors in understanding the relationship between parent and child obesity. Given the growing epidemic of childhood obesity, it is clearly important to conduct further research examining parental influences on eating and weight gain from infancy through adolescence for preterm infants as well as those born at term.

## Abbreviations

ATVV: Auditory, tactile, visual, and vestibular shocking simulation
CA: Corrected age
H-HOPE: Hospital to home transition-optimizing premature infant’s environment
NCAST: Nursing child assessment satellite training

## References

[1] Barnard KE, Hammond MA, Booth CL, Mitchell SK, Spieker SJ, Morrison FJ (1989). Measurement and meaning of parent–child interaction. Applied developmental psychology.

[2] Centers for Disease Control and Prevention. (2015b). “Overweight and Obesity.” Retrieved from. http://www.cdc.gov/obesity/data/index.html. Accessed Jan 2016.

[3] Leddy MA, Power ML, Schulkin K (2008). The impact of maternal obesity on maternal and fetal health. Rev Obstet Gynecol.

[4] Baughcum A, Powers S, Johnson SB (2001). Maternal feeding practices and beliefs and their relationships to overweight in early childhood. Dev Behav Pediatr.

[5] Flegal KM, Carroll MD, Kit BK (2012). Prevalence of obesity and trends in the distribution of body mass index among US adults, 1999–2010. JAMA.

[6] Goldenberg R, Culhane J, Iams K (2008). Epidemiology and causes of preterm birth. Obstetrics.

[7] Forsyth BW, Canny PF (1991). Perceptions of vulnerability 3 1/2 years after problems of feeding and crying behavior in early infancy. Pediatrics.

[8] Ong KK (2006). Size at birth, postnatal growth and risk of obesity. Horm Res Paediatr.

[9] Allen E, Manuel J, Legault C (2004). Perception of child vulnerability among mothers of former premature infants. Pediatrics.

[10] Medoff-Cooper B (2005). Nutritive sucking research: from clinical questions to research answers. J Perinatal Neonatal Nurs.

[11] Medoff-Cooper B, McGrath JM, Bilker W (2000). Nutritive sucking and neurobehavioral development in preterm infants from 34 weeks PCA to term. Am J Maternal Child Nurs.

[12] Medoff-Cooper B, Rankin K, Li Z (2015). Multisensory intervention for preterm infants improves sucking organization. Adv Neonatal Care.

[13] Bakeman R, Brown JV (1980). Early interaction: consequences for social and mental development at three years. Child Dev.

[14] Amir LH, Donath S (2007). A systematic review of maternal obesity and breastfeeding intention, initiation and duration. BMC Pregnancy Childbirth.

[15] Culley BS, Perrin EC, Chaberski MJ (1989). Parental perceptions of vulnerability of formerly premature infants. J Pediatr Health Care.

[16] Estroff DB, Yando R, Burke K (1994). Perceptions of preschoolers’ vulnerability by mothers who had delivered preterm. J Pediatr Psychol.

[17] Perrin EC, West PD, Culley BS (1989). Is my child normal yet? Correlates of vulnerability. Pediatrics.

[18] Forcada-Guex M, Pierrehumbert B, Borghini A (2006). Early dyadic patterns of mother–infant Interactions and outcomes of prematurity at 18 months. Pediatrics.

[19] Strauss R, Knight J (1999). Influence of the home environment on the development of obesity in children. Pediatrics.

[20] Obesity in pregnancy. Committee Opinion No. 549. American College of Obstetricians and Gynecologists. Obstet Gynecol. 2013;121:213–217. https://www.ncbi.nlm.nih.gov/pubmed/23262963. Accessed Jan 2016. 10.1097/01.aog.0000425667.10377.60 23262963

[21] Sobal J, Stunkard A (1989). Socioeconomic status and obesity: a review of the literature. Psychol Bull.

[22] Pulvers KM, Lee R, Kaur H (2004). Development of a culturally relevant body image instrument among urban African Americans. Obes Res.

[23] Pridham K, Steward D, Thoyre S (2007). Feeding skill performance in premature infants during the first year. Early Hum Dev.

[24] White-Traut R, Norr K (2009). An ecological model for premature infant feeding. J Obstet Gynecol Neonatal Nurs.

[25] White-Traut RC, Rankin KM, Yoder JC, et al. Influence of H-HOPE intervention for premature infants on growth, feeding, progression and length of stay during initial hospitalization. J Perinatol. 2015;1–6. http://www.nature.com/jp/journal/v35/n8/abs/jp201511a.html. 10.1038/jp.2015.11 PMC4520757 25742287

[26] White-Traut R, Rankin K, Pham T (2014). Preterm infants’ orally directed behaviors and behavioral state responses to the integrated H-HOPE intervention. Infant Behv Dev.

[27] Centers for Disease Control and Prevention (2015). Preterm birth.

[28] Centers for Disease Control and Prevention (2015). About adult BMI.

[29] Landis JR, Koch GG (1997). The measurement of observer agreement for categorical data. Biometrics.

[30] Bee HL, Barnard KE, Eyres SJ, Gray CA, Hammond MA, Spietz AL, Clark B (1982). Prediction of IQ and language skill from perinatal status, child performance, family characteristics, and mother-infant interaction. Child Dev.

[31] Davidson EC, Hobel CJ (1978). POPRAS: a guide to using the prenatal, intrapartum, postpartum record.

[32] Ross MG, Hobel CJ, Bragonier JR, Bear MB, Bemis RL (1986). A simplified risk-scoring system for prematurity. Am J Perinatol.

[33] White-Traut R, Norr KF, Fabiyi C, Rankin KM, Li Z, Liu L (2013). Mother–infant interaction improves with a developmental intervention for mother–preterm infant dyads. Infant Behav Dev.

[34] Sumner GA, Spietz A. NCAST caregiver/parent-child interaction feeding manual. Seattle: NCAST Publications; 1995.

[35] National Center for Health Statistics in collaboration with the National Center for Chronic Disease Prevention and Health Promotion (2000). https://www.cdc.gov/growthcharts/data/set1clinical/cj41l017.pdf. Accessed Jan 2016.

[36] Baker JL, Michaelsen KF, Sorensen TI (2007). High prepregnant body mass index is associated with early termination of full and any breastfeeding in Danish women. Am J Clin Nutr.

[37] Kugyelka JG, Rasmussen KM, Frongillo EA (2004). Maternal obesity is negatively associated with breastfeeding success among Hispanic but not black women. J Nutr.

[38] Mok E, Multon C, Piguel L (2008). Decreased full breastfeeding, altered practices, perceptions, and infant weight change of prepregnant obese women: a need for extra support. Pediatrics.

[39] Ehrenthal D, Wu P, Trabulsi J (2016). Differences in the protective effect of exclusive breastfeeding on child overweight and obesity by mother’s race. Matern Child Health J.

[40] Rogers SL, Blissett J (2017). Breastfeeding duration and its relation to weight gain, eating behaviours and positive maternal feeding practices in infancy. Appetite.

[41] Puhl RM, Heuer CA (2009). The stigma of obesity: a review and update. Obesity.

[42] Kang R, Barnard K, Hammond M (1995). Preterm infant follow-up project: a multi-site field experiment of hospital and home intervention programs for mother and preterm infants. Public Health Nurs.

